# Metabolic plasticity improves lobster’s resilience to ocean warming but not to climate-driven novel species interactions

**DOI:** 10.1038/s41598-022-08208-x

**Published:** 2022-03-15

**Authors:** Michael Oellermann, Quinn P. Fitzgibbon, Samantha Twiname, Gretta T. Pecl

**Affiliations:** 1 Aquatic Systems Biology Unit, TUM School of Life Sciences, Technical University of Munich, Weihenstephan, Germany; 2 Institute for Marine and Antarctic Studies, University of Tasmania, Hobart, Australia

**Keywords:** Ecophysiology, Ecology, Physiology, Metabolism, Respiration, Zoology, Animal physiology, Climate change, Ocean sciences, Marine biology

## Abstract

Marine species not only suffer from direct effects of warming oceans but also indirectly via the emergence of novel species interactions. While metabolic adjustments can be crucial to improve resilience to warming, it is largely unknown if this improves performance relative to novel competitors. We aimed to identify if spiny lobsters—inhabiting a global warming and species re-distribution hotspot—align their metabolic performance to improve resilience to both warming and novel species interactions. We measured metabolic and escape capacity of two Australian spiny lobsters, resident *Jasus edwardsii* and the range-shifting *Sagmariasus verreauxi,* acclimated to current average—(14.0 °C), current summer—(17.5 °C) and projected future summer—(21.5 °C) habitat temperatures. We found that both species decreased their standard metabolic rate with increased acclimation temperature, while sustaining their scope for aerobic metabolism. However, the resident lobster showed reduced anaerobic escape performance at warmer temperatures and failed to match the metabolic capacity of the range-shifting lobster. We conclude that although resident spiny lobsters optimise metabolism in response to seasonal and future temperature changes, they may be unable to physiologically outperform their range-shifting competitors. This highlights the critical importance of exploring direct as well as indirect effects of temperature changes to understand climate change impacts.

## Introduction

By the end of this century our oceans will likely be, on average, 3.5 °C warmer (relative to 1870–1899, RCP8.5^1^). Local warming can be even more extreme, due to heat waves^2^, changing ocean currents^3^, or cyclic weather patterns^4^. Such warming hotspots show rapid change of ecosystems, characterised by altered species abundance, biodiversity decline and local extinctions^5,6^. Species persistence will depend on their ability to acclimate or adapt rapidly^7^, or alternatively by ‘escaping’ to geographically track suitable temperatures poleward^8–10^. As a result, species are now re-distributing globally, particularly in our ocean, where species ranges shift up to six times faster than on land (5.9 vs. 1.1 km per year^9,11^). However, due to differences in physiological tolerance, species traits, behaviour, habitat availability, adaptive capacity or access to microclimates, species may shift at different rates leading to disassembly of existing communities or emergence of novel biotic interactions^9,10^. Shifting to keep pace with preferred temperatures, or conversely, maintaining presence in an existing part of a specie’s distribution, may be further complicated by changing predation or competition pressures as result of range-shifting species^12–14^. For many species, acclimation or adaptation will increase resilience to these challenges and be key for their survival^7^.

Niche shifts via physiological adjustments in response to environmental change (i.e. physiological plasticity or acclimation) are a critical and rapid mechanism by individuals to improve resilience to increasing temperatures^15–17^ and reduce extinction risk^7^. Energy metabolism plays an important role in this context^18^. It powers fundamental processes such as growth, locomotion, or reproduction that require energy in the form of ATP produced either aerobically or anaerobically. Aerobic metabolism is far more efficient (~ 36 ATP/glucose molecule) and consequently the predominant process in most organisms to power sustained activities^19^. Performance declines due to temperature changes are thus frequently compensated by plastic adjustments of aerobic pathways, characterized by shifts in e.g. metabolic rate^20^ or mitochondrial function^21,22^. On the other hand, anaerobic sources of energy, such as free ATP, muscle phosphocreatine, or ATP derived from glycolysis^23,24^, are quickly exhausted but can release energy more rapidly. This supports short, strong bursts of activity, and is critical to survival e.g. when escaping from predators^25^. Consequently, failure to sustain or adjust both aerobic and anaerobic energy metabolism may not only impair an animal`s performance but directly affect their survival in increasingly warmer waters.

A largely unexplored aspect has been how such metabolic plasticity shapes outcomes of species interactions, particularly between resident and range-shifting species^8^. Most marine animals are unable to regulate their body temperature (i.e., they are ectothermic) and perform well only within a limited range of temperatures. Temperature, therefore not only limits species’ geographic distribution^26^ but also regulates how ectothermic species perform relative to each other, depending on the individual shape and overlap of their thermal windows^18,27^. Physiological plasticity can shift thermal niches and consequently the outcomes of direct interactions, such as competition^28^, resulting in the dominance of the resident or the range-shifting species. Given this, it is essential to understand species’ capacity for physiological plasticity to predict their future distributions and outcomes of biotic interactions^8^.

## Lobsters in a warming and range-shift hotspot

Lobsters are key to biodiverse underwater ecosystems, both as prey and predators^29^, and improve resilience to climate-driven catastrophic ecosystem shifts, by consuming invasive barren-forming sea urchins^30,31^. At the same time, lobsters are themselves highly vulnerable to climate warming, with local temperatures already exceeding thermal tolerance limits leading to local population declines^32–34^. This has not only consequences for the ecosystems they support but also their supply as a valuable resource to coastal and indigenous communities^35,36^ with a global annual market worth 3.3 billion USD^37^.

**Figure 1:**
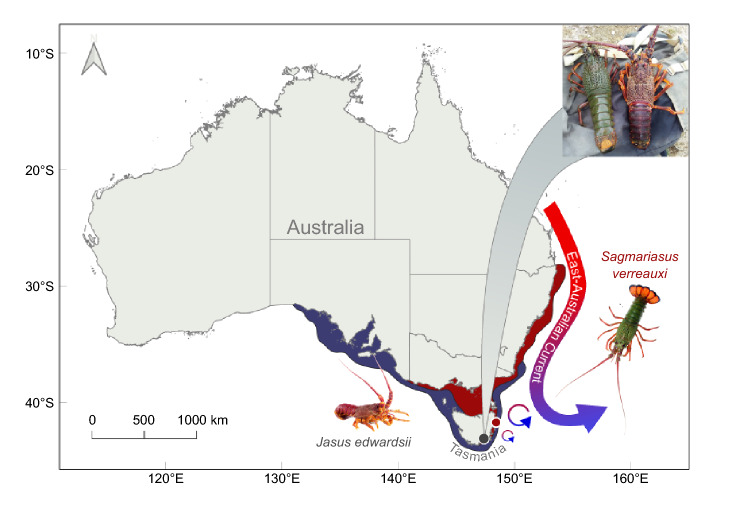
Species distribution map of the southern rock lobsters (*J. edwardsii*, blue) and the subtropical eastern rock lobster (*S. verreauxi*, red). The East Australian Current carries tropical warm water southwards leading to increasingly warmer waters in South-East Australia and Tasmania. An increasing number of eastern rock lobsters now co-occur with resident southern rock lobsters as they track the warming waters (photo taken on 18.01.2020 in Hobart area, source: www.redmap.org.au/sightings/3654/). Catch location marked by coloured circles. Image courtesy of Craig Mostyn Group, Seafood New Zealand and Andrew Cables.

With at least ten species, Australia hosts a rich diversity of spiny lobsters^38^. However, warming threatens this diversity, with lobster distributions predicted to contract by 40–100%^39^, particularly in South–East Australia, where waters are heating up 3–4 times faster than the global average^3,40^. This warming trend has led to dozens of species shifts from coastal mainland Australia to the cold-temperate waters of Tasmania^41–45^. Among them is the eastern rock lobster *Sagmariasus verreauxi*, the world’s largest spiny lobster reaching up to 20 kg and 70 cm total length^38,46^, that has become increasingly abundant in areas previously occupied exclusively by the resident southern rock lobster *Jasus edwardsii*^42^ (Fig. 1).

Southern rock lobsters cannot evade warming trends due to the lack of coastal habitat further south^40^ (Fig. 1) and by 2070 may face up to 21.5 °C on average during summer in South–East Tasmania (high emission scenario A1F1 in Pecl *et* al. 2009^47^). These warm temperatures will exceed southern rock lobster thermal optima for growth (20.6 °C^48^), feed efficiency (19.3 °C^48^), and metabolic scope (19.6 °C^49^). Moreover, an increasing number, intensity and duration of heat waves will add up to an extra 2 °C of warming^50^, posing an acute risk to physiological functioning (e.g. declining oxygen consumption > 22 °C^48^), declining cellular energy production (> 25 °C^51^) and survival of *J. edwardsii* (> 23.3–24 °C^48,52^). Any additional stress by competition such as reduced quantity or quality of diet^53^, disease^54^, loss of shelter^55^ or increased predation^56^, may be detrimental to the survival of local populations of *J. edwardsii*. Yet, if *J. edwardsii* can dynamically adjust thermal windows of essential processes such as energy metabolism, it may increase resilience to the dual climate-driven pressure of warming and a novel range-shifting competitor.

Although the thermal ecology of *J. edwardsii* and *S. verreauxi* has been relatively well studied^49,51,57^, it remains unknown whether metabolic plasticity changes their resilience to warming and the relative performance between the species.

This study aimed to identify if resident and range-shifting, co-occurring spiny lobster species inhabiting an Australian warming hotspot: (1) adjust their metabolism in response to seasonal and forecasted temperature changes and (2) if this leads to a relative shift of metabolic performance between the two species.

Measurements of oxygen consumption rates showed that both spiny lobster species decrease their standard metabolic rate with increasing acclimation temperature while sustaining their scope for aerobic metabolism. However, resident *J. edwardsii* showed decreased anaerobic escape capacity at 21.5 °C acclimation temperature and failed to match the metabolic capacity of the range-shifting lobster. Although, metabolic plasticity aids resident lobsters to cope with direct effects of ocean warming, it does not suffice coping with indirect warming impacts such as novel range-shifting competitors.

## Results

### Thermal plasticity of aerobic metabolism

**Figure 2:**
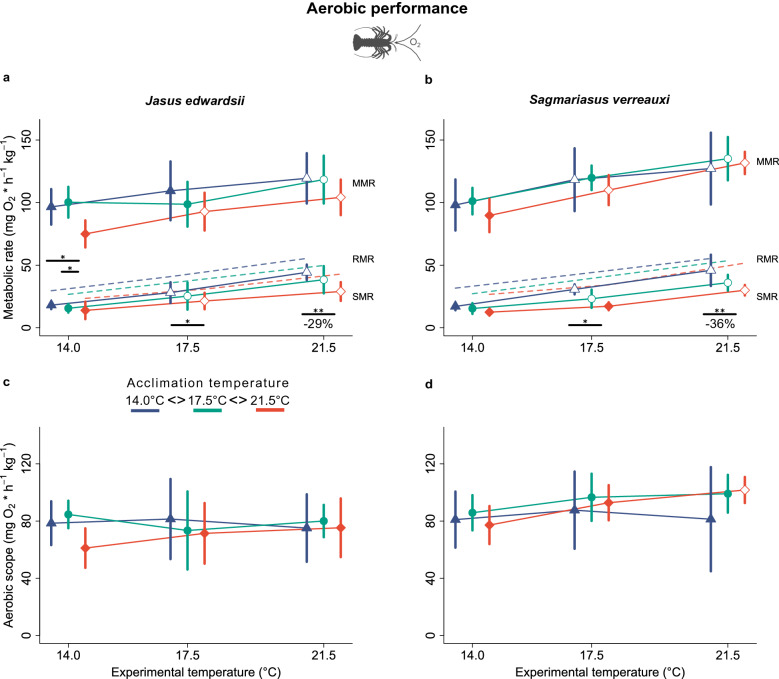
Change of (**a**,**b**) standard-, routine-, and maximum metabolic rate and (**c**,**d**) aerobic scope in response to acute and chronic temperature changes in comparison between *J. edwardsii* and *S. verreauxi*. Data presented as means ± 95% C.I., *n* = 6. Colours and x-axis offsets indicate thermal acclimation to 14.0 °C (blue triangles), 17.5 °C (green circles) and 21.5 °C (red squares). Underscored asterisks below data points indicate significant differences between acclimation temperatures (* < 0.05, ** < 0.01). Numbers below lines show the percentage decrease of SMR from 14 to 21.5 °C acclimation temperature. Open circles indicate a significant difference relative to the lowest experimental temperature for each acclimation group.

Oxygen consumption measurements following exhaustion experiments showed that the resident and the range shifting lobster—inhabiting the same ocean warming hotspot—both decrease their standard metabolic rate^58^ with increasing acclimation temperature, while sustaining their aerobic scope (Fig. 2a–d). Following a minimum 8 weeks of thermal acclimation to current average—(14.0 °C), current summer—(17.5 °C) and future summer—(21.5 °C) habitat temperatures, standard metabolic rates were 29% lower in warm-acclimated (21.5 °C) *J. edwardsii* (29.0 mg O_2_ h^−1^ kg^−1^ [21.4–36.5] at 21.5 °C versus 44.3 mg O_2_ h^−1^ kg^−1^ [38.1–50.5] at 14.0 °C, *n* = 6) and 36% lower in warm-acclimated *S. verreauxi* (30.0 mg O_2_ h^−1^ kg^−1^ [25.8–34.2] at 21.5 °C versus 45.9 mg O_2_ h^−1^ kg^−1^ [33.4–58.3] at 14.0 °C, *n* = 6) compared to their cold acclimated (14 °C) counterparts at an experimental temperature of 21.5 °C (Fig. 2a–b, Supplementary Table S3). This was due to a lower thermal increase of standard metabolic rates with increasing experimental temperatures in warm-acclimated lobsters (Table 1). This difference in standard metabolic rate among acclimation groups increased successively from 14 to 21.5 °C experimental temperature (Fig. 2a–b, Table 2). Unlike standard metabolic rates, which showed a relatively high thermal sensitivity, maximum metabolic rates increased only moderately with experimental temperatures in both species (Table 1) and were more similar among acclimation groups (Fig. 2a–b). Only warm-acclimated (21.5 °C) *J. edwardsii* showed a 10–20% lower maximum metabolic rate compared to the 17.5 °C and 14 °C acclimation groups at 14 °C experimental temperature (Fig. 2a, Table 2). Interestingly, aerobic scope did not differ among acclimation groups in either of the two species (Fig. 2c-d), except for a minor trend of decreased aerobic scope for warm acclimated *J. edwardsii* at 14 °C (Fig. 2c, Table 2). Furthermore, thermal sensitivity of aerobic scope was low in both species and among acclimation groups (Table 1). Only warm-acclimated *S. verreauxi* showed an increased aerobic scope from 14 to 21.5 °C experimental temperature (Fig. 2d), largely due to a more pronounced increase in maximum metabolic rate relative to standard metabolic rate (Fig. 2a). Factorial aerobic scope, which expresses the ratio between maximum metabolic rate and standard metabolic rate, decreased largely towards warmer experimental temperatures and increased significantly towards warm-acclimation for both species (Table 2, Supplementary Table S2).

**Table 1:** Thermal coefficients (Q_10_) for standard metabolic rate (SMR), maximum metabolic rate (MMR) and aerobic scope in comparison between *J. edwardsii* and *S. verreauxi*.

Species	Trait	14.0 °C	17.5 °C	21.5 °C
*Jasus edwardsii*	SMR	3.3	3.3	2.7
	MMR	1.3	1.2	1.6
	Aerobic scope	0.9	0.9	1.3
*Sagmariasus verreauxi*	SMR	3.7	3.1	3.2
	MMR	1.4	1.5	1.7
	Aerobic scope	1.0	1.2	1.4

Q_10_ were calculated from data means covering the experimental temperature range from 14.0 to 21.5 °C.

**Table 2:** Final linear- (LME) or generalised mixed effect models (GLMM) selected for each response variable.

Variable	Model type	Final model
SMR	GLMM	T_Experiment_ × T_Acclimation_ + Sex + (1|Animal ID)
MMR	LME	T_Experiment_ × T_Acclimation_ × Species + Body mass + (1|Animal ID)
Aerobic scope	LME	T_Experiment_ × T_Acclimation_ + Species + (1|Animal ID)
Factorial aerobic scope	LME	T_Experiment_ + T_Acclimation_ + (1|Animal ID)
EPOC	LME	T_Experiment_ × T_Acclimation_ × Species + (1|Animal ID)
Recovery time	LME	T_Experiment_ + T_Acclimation_ + (1|Animal ID)
Recovery rate	LME	T_Experiment_ × T_Acclimation_ + (1|Animal ID)
Escape speed (cm s^−1^)	LME	T_Experiment_ + Species + (1|Animal ID)
Total escapes	LME	Species + Body mass + (1|Animal ID)

*SMR* standard metabolic rate, *MMR* maximum metabolic rate, *EPOC* excess post-exercise oxygen consumption rate, *T* Temperature.

### Anaerobic escape performance

**Figure 3:**
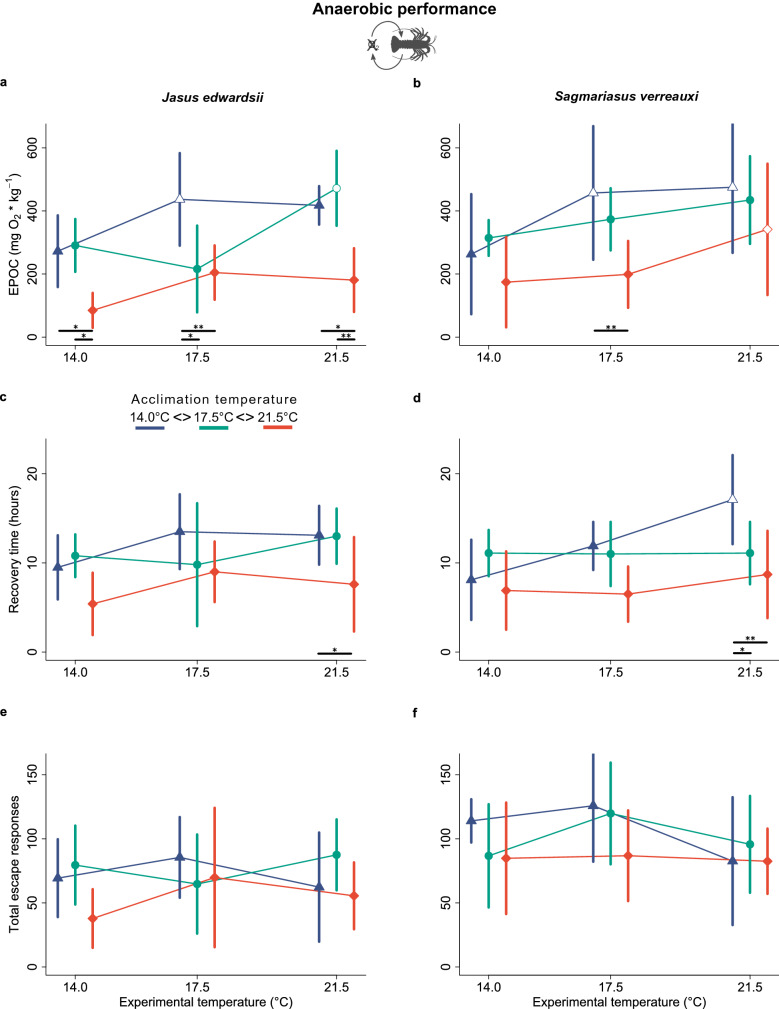
Change of (**a**,**b**) Excess post-exercise oxygen consumption rate (EPOC), (**c**,**d**) time to recover to pre-exercise metabolic rates and (**e**,**f**) total number of escape response in response to acute and chronic changes of temperature in comparison between *J. edwardsii* and *S. verreauxi*. Data presented as means ± 95% C.I., *n* = 6. Colours and x-axis offsets indicate thermal acclimation to 14.0 °C (blue triangles), 17.5 °C (green circles) and 21.5 °C (red squares). Underscored asterisks below data points indicate significant differences between acclimation temperatures (* < 0.05, ** < 0.01). Open circles indicate a significant difference relative to the lowest experimental temperature for each acclimation group.

Simultaneous to the aerobic component supporting exhaustive exercise in spiny lobsters, we assessed the anaerobic component during respirometry expressed as excess post-exercise oxygen consumption rate (EPOC), which represents the additional oxygen required to return depleted anaerobic energy stores, pH and lactate levels during recovery back to pre-exercise levels^59,60^. Warm-acclimation to 21.5 °C largely reduced EPOC by 59% in *J. edwardsii* (156.7 mg O_2_ kg^−1^ [109.9–203.6], *n* = 18) compared to lobsters acclimated to 14 °C (375.6 mg O_2_ kg^−1^, [311.1–440.1], *n* = 18), when averaged across experimental temperatures (Fig. 3a, Table 2). EPOC decreased similarly by 36% in warm-acclimated *S. verreauxi* (238.3 mg O_2_ kg^−1^, [155.5–321.0], *n* = 18) compared to cold-acclimated conspecifics (398.4 mg O_2_ kg^−1^, [291.7–505.1], *n* = 18) but was less pronounced and only significant at 17.5 °C experimental temperature (Fig. 3, Supplementary Table S2). Recovery time, which represents the time lobsters require to replenish depleted anaerobic energy stores and return to pre-exercise oxygen consumption rates (*M*O_2_), was on average 4.7 and 5 h shorter in warm-acclimated (21.5 °C) *J. edwardsii* (12.0 h [10.1–14.0] at 21.5 °C versus 7.3 h [5.2–9.4] at 14.0 °C, *n* = 18) and *S. verreauxi* (12.4 h [9.7–15.1] at 21.5 °C versus 7.4 h [5.4–9.4] at 14.0 °C, *n* = 18) respectively compared to cold acclimated (14 °C) individuals (Fig. 3c,d, Table 2, Supplementary Table S2). This was most apparent at an experimental temperature of 21.5 °C in both species (Fig. 3c,d). Although there was no significant effect of thermal acclimation on recovery rates (Supplementary Table S2), warm-acclimated *J. edwardsii* tended to replenish 32.1% less oxygen per hour than cold acclimated conspecifics (22.0 mg O_2_ h^−1^ kg^−1^ [18.3–25.7] at 21.5 °C versus 32.4 mg O_2_ h^−1^ kg^−1^ [28.1–36.8] at 14.0 °C, *n* = 18).

Further, escape performance—expressed as the total number of tail flips lobsters performed during the exhaustion experiments—did not change significantly with thermal acclimation in both lobster species (Fig. 3e–f, Table 2) although there was a tendency for warm-acclimated *J. edwardsii* tended to perform 52% and 37% less tail flips at 14.0 °C and 21.5 °C experimental temperature respectively in comparison to 17.5 °C acclimation temperature (Fig. 3e). Escape speed was unaffected by thermal acclimation in both lobsters irrespective if expressed in absolute terms (Table 2) or relative to size (Supplementary Files S2). In addition, correlation analysis showed a significant relationship between tail flips and EPOC for *J. edwardsii* (Pearson: *r*_(51)_ = 0.39, *p* = 0.004) but not *S. verreauxi* (Pearson: *r*_(51)_ = 0.23, *p* = 0.098). Correlation between size dependent escape speed (carapace length s^−1^) and EPOC was significant for both *J. edwardsii* (Pearson: *r*_(51)_ = 0.34, *p* = 0.013) and *S. verreauxi* (Pearson: *r*_(51)_ = 0.28, *p* = 0.046).

### Resident versus range-shifting lobster

**Figure 4:**
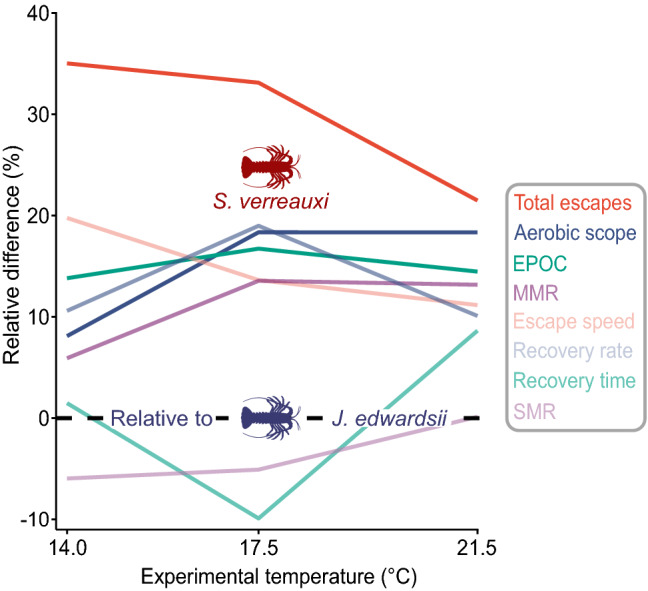
Relative divergence (%) of metabolic traits and escape capacity of range expanding *S. verreauxi* (lines) relative to resident *J. edwardsii* (dashed line) across annual average-, current summer- and future summer (2070) sea water temperatures in South–East Australia. Data were pooled for each acclimation temperature.

In addition to the effects of thermal acclimation, there were marked performance differences between the temperate *J. edwardsii* resident to Tasmania and the subtropical, range-shifting *S. verreauxi*. Most noticeable, on average, aerobic scope was 8–18% higher in *S. verreauxi* (89.2 mg O_2_ × h^−1^ kg^−1^ [83.3–94.6], *n* = 54), compared to *J. edwardsii* (75.6 mg O_2_ × h^−1^ kg^−1^ [70.3–81.0], *n* = 54, Supplementary Table S2) particularly towards warmer experimental temperatures (≥ 17.5 °C, Fig. 4, Table 2). This was due to a 6–14% larger maximum metabolic rate in *S. verreauxi* (114.6 mg O_2_ × h^−1^ kg^−1^ [108.5–120.6], *n* = 54) compared to *J. edwardsii* (101.7 mg O_2_ × h^-1^ kg^−1^ [96.1–107.2], *n* = 54), while there were no apparent differences between species for standard metabolic rates (Fig. 4, Table 2).

Performance indicators of anaerobic metabolism, including EPOC, recovery time and recovery rate did not differ significantly between both species, despite *S. verreauxi* tending towards 14–17% higher EPOC and 10–19% faster recovery rates (Figs. 3a–d,  4, Supplementary Table S2). However, *S. verreauxi* escaped 11–20% faster and performed 22–35% more tails flips compared to *J. edwardsii* from 14 to 21 °C experimental temperatures (Fig. 4, Supplementary Table S2).

## Discussion

Marine organisms are highly vulnerable to climate warming, not only by being exposed to (potentially) critically high temperatures but also indirectly by a global climate-driven redistribution of species that may bring novel prey, predators, and competitors^9^. Phenotypic plasticity is one vital mechanism of species to reduce climate-driven temperature stress^15^ and extinction risk^7^, yet whether this improves resilience to novel competitors remains unknown.

In this study we found that both a resident and a range-shifting spiny lobster dynamically adjust aerobic metabolism to sustain physiological performance in response to seasonal and forecasted temperature changes. However, at future summer temperatures, resident lobsters lose anaerobic escape capacity and fail to match the metabolic performance of range-shifting lobsters. Resident Tasmanian spiny lobsters are thus mal-equipped to cope with the dual pressures of warming and novel competitors.

### Metabolic plasticity

Plastic adjustments (phenotypic plasticity) of metabolic rate are a common means for organisms to balance performance and thermodynamic increases of energy expenditure when temperatures rise^15^. This is particularly vital for organisms inhabiting ocean warming hotspots, such as South–East Australia, which is heating up to 3–4 times faster than the global average^3,40^. We found that resident spiny lobster *J. edwardsii* can respond to such drastic changes, by dynamic shifts of standard metabolic rate, which reduces its basic energy needs by 29% at projected summer temperatures of 21.5 °C (by 2070) compared to cold-acclimated lobsters (14 °C, Fig. 2a). Without this crucial adjustment, basic metabolic energy demand would increase and require lobsters to invest more time, energy, and risk to find prey to fuel this extra demand as well as trigger risky behavioural responses to predators^61^.

Adjusted standard metabolic rates further aided to sustain aerobic scope (the range between standard- and maximum metabolic rate) in both lobster species at warmer temperatures (Fig. 2c–d). This is because, given the lack of thermal compensation and a lower thermal increase of maximum metabolic rate (Table 1), aerobic scope would decline towards warmer temperatures without the observed reduction of standard metabolic rate. Such an adjustment aids both lobster species, particularly cold-temperate *J. edwardsii*, to sustain full capacity for other non-maintenance related oxygen-fuelled activities up to 21.5 °C projected summer temperatures. This may include feeding, digestion, migration, social interactions, or highly stressful events like moulting, which can exhaust the full aerobic scope of individual lobsters (personal observation, Supplementary Fig. S1).

As for the two spiny lobster species in this study, metabolic plasticity is an important means for several other species to cope with temperature changes, particularly for aquatic ectotherms, such as molluscs and fish, but also other decapod crustaceans^15,62^. Yet, the underlying patterns are less well understood. Plastic standard metabolic rates but more rigid maximum metabolic rate observed in *J. edwardsii* and *S. verreauxi* (Fig. 2a–b), have been found in European perch too, and was coined as “plastic floors and concrete ceilings”^63^. Although not being a universal strategy^64^, this indicates that, like perch, spiny lobsters prioritise adjustments of standard metabolic rate over maximum metabolic rates, which has the dual benefit of lowering maintenance costs while maintaining scope for aerobic activities. A further increase of aerobic scope at warmer temperatures is either not critical to sustain daily activities or further rate increases of maximum metabolic rate are limited, by exhausted mitochondrial densities or capacities^51^, cardiac performance^65^, ventilatory oxygen extraction, or blood oxygen transport^66–68^.

On the other hand, alternative mechanisms may cause the observed depression of standard metabolic rate. For example, variation of organ mass can be an effective way to modify basic energy consumption^19^, particularly in case of highly active tissues such as hearts or liver, which can explain up to 38% of standard metabolic rate variation (e.g., European eel^69^). Changes of mitochondrial activities and densities are additional means to modify energy consumption, as the case for American lobsters where the activity of mitochondria’s key enzyme citrate synthase declined by 35% in tail muscle under combined exposure to high temperatures and CO_2_^70^. Temperature induced changes in mitochondrial function, such as differential substrate use or improved efficiency of oxidative phosphorylation can further optimise energy consumption^21,71^. Lobsters can also enlarge muscle fibres—at least during development—to lower surface to volume ratio and consequently reduce energetic costs to maintain membrane potential^72,73^. This however is limited by larger intracellular diffusion distances limiting oxygen flux^73^. Irrespective of the mechanism at work, the observed plasticity of standard metabolic rate aids resident *J. edwardsii* to reduce fundamental energetic costs, which increases resilience to future warming trends particularly for marginal populations at the warmer trailing edge^8^.

### Anaerobic escape performance

Spiny lobsters escape from predators or other threats by powerful tail-flips, largely fuelled by anaerobic energy metabolism^74,75^. Exhaustive escape trials in this study revealed that anaerobic capacity, measured indirectly as excess post-exercise oxygen consumption rate (EPOC), declined steeply by 59% in warm-acclimated (21.5 °C) *J. edwardsii* (Fig. 3a). This was further supported by fewer escape attempts (tail flips), aligning closely with decreased EPOC (Fig. 2e). Therefore, although *J. edwardsii’*s aerobic performance remained stable, anaerobic performance declined considerably, compromising its endurance to escape repeated threats when climate warming or heatwaves increase ocean temperatures to 21.5 °C or higher in South–East Australia.

But why would *J. edwardsii’*s escape performance decline when ocean temperatures rise? In crustaceans, including spiny lobsters, burst muscle contractions are fuelled anaerobically^75^, particularly in large white muscle fibres^74^. Initial bursts compose rapid tail flips fuelled by intracellular arginine phosphate pools, followed by slower less powerful bursts supported by anaerobic glycogenolysis, generating ATP from stored glycogen^76,77^. Initial arginine phosphates stores did not seem limiting in warm-acclimated *J. edwardsii*, as it performed initial escape bursts at full speed. However, its, by trend, lower numbers of repeated bursts compared to cold-acclimated conspecifics (Fig. 3e), suggests that instead anaerobic glycogenolysis was limiting. This could be explained by lower glycogen stores or decreased glycolytic enzyme activity induced by chronic warming, yet current evidence is lacking, and abdominal glycogen rather seems to be enhanced in warm-acclimated European lobsters (*Homarus gammarus*)^78^. Alternatively, warmer temperatures may reduce *J. edwardsii’*s ability to buffer lactate and protons accumulated during anaerobic (glycolytic) ATP production, as is the case for American lobsters, which have limited capacity to buffer low haemolymph pH with HCO_3_^−^ and ammonia at higher temperature and *p*CO_2_^70^. The resulting drop in intracellular pH would then inhibit glycolytic enzymes and limit anaerobic power supply^79^.

Following the anaerobic power phase, lobsters eventually enter a slow oxygen-consuming (aerobic) recovery phase (i.e., EPOC) to return glycogen, pH, and lactate to pre-exercise levels^59^. Here, warm-acclimated *J. edwardsii* recovered 4.7 h faster than cold-acclimated conspecifics (Fig. 2c), mirroring the lower oxygen depth accumulated during the limited anaerobic power phase. Yet interestingly, warm-acclimated *J. edwardsii* tended to have 32.1% lower recovery rates (i.e., oxygen replenished per hour) than cold-acclimated animals, indicating reduced capacity for aerobic recovery, due to e.g., reduced mitochondrial enzymes activity or densities^70^. This may link to the observed reduction in basic energy consumption and would be an interesting path for further investigation together with spiny lobster’s capacity to buffer lactate and pH at future ocean warming scenarios.

As a result, if ocean temperatures continue to rise till 21.5 °C, *J. edwardsii* will sustain its ability to escape from predator attacks, however, only if they occur at low frequency. At higher frequencies, *J. edwardsii* will quickly exhaust and be highly vulnerable to vigorous predators. Its sensitivity may increase even further in face of additional disturbances, such as high fishing pressure^80^, disease^81^, or lack of shelter in impoverished habitats^82,83^.

### Resident versus range shifting lobsters

Although we found that *J. edwardsii* improves resilience to future ocean warming, by energy-conserving metabolic adjustments, this did not improve physiological performance relative to a novel range-shifting competitor, the eastern rock lobster *S. verreauxi*. This subtropical species increasingly co-occurs in the resident temperate habitat of *J. edwardsii* (Fig. 1) and will likely compete for shelter and/or food^84,85^, particularly in resource impoverished localities^86^, such as recently formed urchin-dominated barren habitats^83^. In addition to the fact that *S. verreauxi* grows faster and much larger^46^, we found that it consistently matches or exceeds physiological and escape performance of *J. edwardsii* between 14 and 21 °C, which included higher maximum metabolic rate, aerobic scope, escape frequency and speed (Fig. 4, Table 2). Even at, for *S. verreauxi*, relatively cold temperatures of 14 °C, which is far below its optimal temperatures for growth (21.2 °C^57^) or aerobic scope (24.9 °C^34^), it performed 35% more tail-flips and escaped 20% faster than *J. edwardsii* (Fig. 4). Furthermore, despite their different thermal origins, *S. verreauxi’*s standard metabolic rate closely matched that of *J. edwardsii* between 14 and 21.5 °C, and equally decreased with warm acclimation conserving 36% of metabolic energy (Fig. 2b). These findings are in line with previous results of higher maximum metabolic rate and aerobic scope but similar standard metabolic rates between 22 and 24 °C for *S. verreauxi* puerulus larvae and juveniles compared to *J. edwardsii*^49^. This indicates that various life stages of *S. verreauxi* will outperform *J. edwardsii* in situations where physiological capacities become critical, particularly at current and future summer temperatures. For instance, during summer, *S. verreauxi* would have a larger metabolic scope to support migration, ranging or feeding^87^, which could become a vital factor if both lobsters increasingly share habitat and resources, particularly in resource-poor habitats such as urchin barrens. Moreover, although, *S. verreauxi’s* escape performance is below or at similar levels of *J. edwardsii’*s at larval and juvenile life stages^49^, our study showed that this relationship inverts once *S. verreauxi* matures. As a result, larger *S. verreauxi* will be better able to fend-off vigorous predators such as Maori octopus (*Octopus maorum*), which may then preferentially target *J. edwardsii* instead, benefiting further expansion of *S. verreauxi*. If such flow-on effects add to negative interactions with other range-shifting species (e.g., *Octopus tetricus*), resident *J. edwardsii* will face increasing risk of range contraction^88^.

Metabolic performance differences have shown to influence competitive outcomes in other species. For example, a 3.2-fold higher routine metabolic rate linked to a three times higher feeding rate and a 6.7 times higher attack coefficient in the invasive Chinese mitten crab *Eriocheir sinensis,* compared to the co-existing native European crayfish *Austropotamobius pallipes*^89^. Similarly, at 9 °C cold-adapted Arctic staghorn sculpin *Gymnocanthus tricuspis* had a lower aerobic scope than sculpins from warmer latitudes, which outcompeted Arctic staghorn sculpin in the search for protective shelter^90^. Both examples indicate that a larger metabolic scope for activity supports competitive dominance. However, given that differences of aerobic scope were much reduced at 4 °C among sculpins, it further highlights the modulating role of temperature. For example, warm-acclimated freshwater crayfish *Cherax destructor* won over cold-acclimated conspecifics, supported by up-regulated mitochondrial ATP production capacity in chelae (pincer) muscle^71^. Further, in case of two co-existing Australian crayfish*,* temperatures markedly changed tail-flip performance, which peaked at different temperatures for each species, benefiting warm-adapted crayfish to better escape predators when temperatures rise^91^. This is in line with our findings, underlining that range-shifting subtropical *S. verreauxi’*s higher aerobic and escape performance, will provide a clear advantage over resident temperate *J. edwardsii* at current and future summer temperatures. This short-coming was not set-off by *J. edwardsii’*s adjustments of standard metabolic rates as range-shifting *S. verreauxi* mirrored this metabolic plasticity.

While this study provided valuable mechanistic insights about spiny lobster’s adaptive capacity to warming and additional impacts by range shifting competitors, it must be noted that inference was drawn from 18 specimens per species, each sampled from a single local population. For example, *S. verreauxi* was collected from a poleward-edge population, which may consist of pioneering specimens with higher cold-tolerance or ability to cope with novel combinations of abiotic and biotic conditions compared to centre or trailing edge populations (Donelson et al. 2019^8^). Additional observation may provide advanced insights as to whether the observed patterns are consistent, or if there are additional physiological phenotypes more or less resilient to ocean warming and novel species interactions (Kroeker and Sanford 2021^18^). Therefore, although, fully factorial physiological experiments like this study are logistically and financially challenging, this study provides a solid basis for future studies to focus efforts on critical traits and assess adaptive potential of spiny lobsters across diverse populations and latitudinal gradients. Further, while this study highlighted critical interactions between physiological performance, temperature, and competition, we stress the need to integrate further important factors driving abundance and species range shift such as fishing pressure^80^, predation^92^, larval recruitment^34^, disease^81^ or habitat loss^93^.

### Conclusion

**Figure 5:**
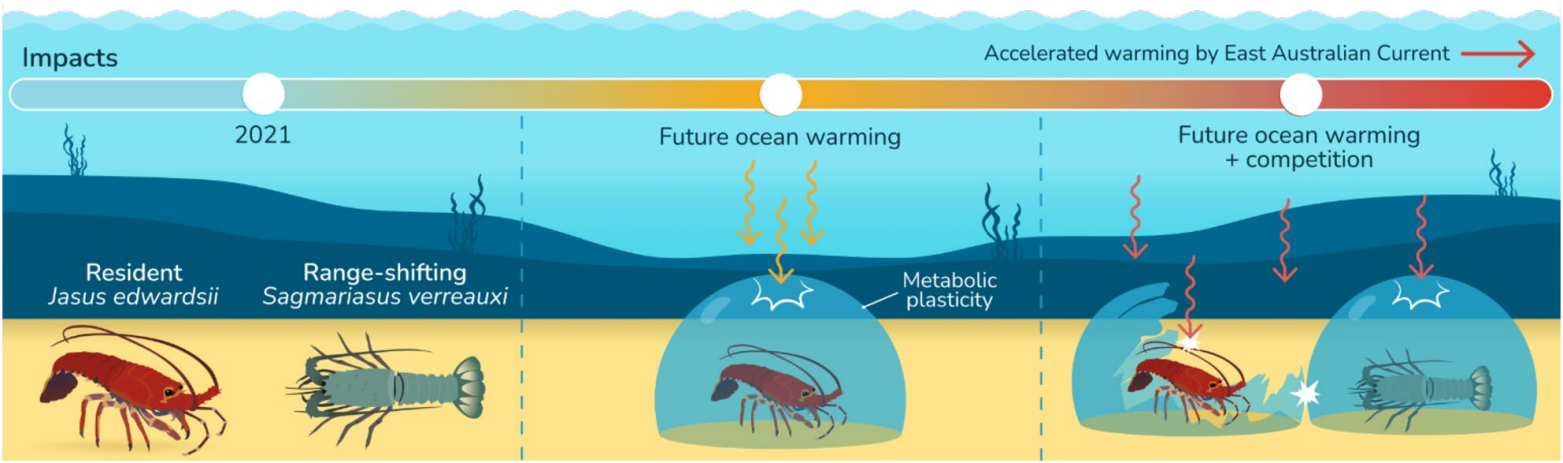
Conceptual diagram, illustrating that metabolic plasticity may aid resident spiny lobsters to resist direct effects of increasing ocean temperatures but not additional indirect pressures brought about by warming such as novel interactions with range-shifting competitors. Image courtesy of Stacey McCormack.

In this study we showed that resident *J. edwardsii* increased its resilience to ocean warming by metabolic plasticity, helping to conserve basic energy consumption and sustain scope for aerobic activities at future summer temperatures. However, this did not aid *J. edwardsii* to overcome the metabolic performance deficits in comparison to the range-shifting spiny lobster *S. verreauxi* and was further set back by reduced anaerobic escape capacities of *J. edwardsii* in response to future summer temperatures. We conclude that resident species like Tasmanian spiny lobsters may be able to cope with the direct effects of increasing ocean temperatures but will struggle to endure additional indirect pressures brought by warming such as novel interactions with range-shifting competitors (Fig. 5). Trends exhibited for American lobsters, where distributions shifted poleward and offshore in response to warming, shell disease and novel invasive species^33,94^ may foreshadow *J. edwardsii’*s future. Yet, the lack of coastal habitat hinders any poleward shift for *J. edwardsii*, stressing the importance to ease environmental and fishing pressures, particularly for northern populations being most exposed to warming and novel species interactions.

## Methods

### Animals

Study animals comprised two species of Australian spiny lobsters. Cold-temperate southern rock lobsters (*J. edwardsii*, body mass 1074.6 g [976.0–1173.2], 12 males, 7 females) were caught from the Taroona marine research reserve at Crayfish point, Tasmania, using baited lobster pots from 5 to 9th February 2018. Subtropical eastern rock lobster (*S. verreauxi*, body mass 1138 g [1054.1–1222.6], 12 males, 7 females) were obtained from commercial rock lobster fishermen (from Triabunna marina, Dover Seafoods and Leale Fishing, St Helens, Tasmania) from August 2017 till February 2018, who caught *S. verreauxi* as bycatch in coastal waters of eastern Tasmania. Lobsters were transported in ice-chilled polystyrene boxes to the IMAS Taroona research facilities, and each species kept separately in two 4000-L fiberglass outdoor tanks, supplied with flow-through seawater till the beginning of acclimation experiments.

Their distribution ranges from southern Victoria, around Tasmania and across South Australia into Western Australia as well as New Zealand waters for *J. edwardsii*, and along the east Australian coastline between Brisbane and the North-East coast of Tasmania, including the northern waters of New Zealand for *S. verreauxi* (Fig. 1). Both species live at depths ranging from 5 to 200 m at the Australian continental shelf^38^. Natural temperature ranges are ~ 10.8–17.5 °C for *J. edwardsii* (Ion Pot, Tasmania^2,95^) and ~ 12–28 °C for *S. verreauxi*^96^. Animal ethics were not required at the time of experimentation.

### Temperature acclimation

Animals were acclimated to three temperatures for at least 8 weeks. Acclimation temperatures were (1) the annual average—(14.0 °C, [14.1–14.2], *n* = 46,764), (2) the average summer—(17.5 °C, [17.4–17.5], *n* = 7,988, for the two warmest months February–March) and (3) the average summer sea water temperature predicted for the East-Coast of Tasmania in 2070 (21.5 °C, predicted temperature increase of 4 °C based on IPCC AR5 scenario RCP8.5 following Hobday 2018 personal communication and the A1FI scenario in Pecl *et* al. 2009^47^). Temperature means were calculated from temperature records at 9 m depth, Ion Pot, Tasmania from 2006 to 2016^2,95^ (data and R script in Supplementary File S1). As only two lobsters could be examined within 48 h, lobsters were acclimated, sequentially in pairs of one lobster for each species. This assured similar acclimation times at the time of the first measurement and further reduced variability introduced by parallel respirometry setups and handling by multiple experimenters. Lobsters were added randomly to acclimation tanks while accounting for balanced body mass distribution between acclimation groups (ANOVA, *F*_(1, 38)_ = 0.78, *p* = 0.384) and species (ANOVA, *F*_(1, 38)_ = 1.16, *p* = 0.289). Although we were unable to obtain equal numbers of females and males, we assured even distribution of sex across species and acclimation groups (Supplementary File S3).

Lobsters were housed in identical coated glass-fibre tanks (W × L × H, in cm, 100 × 100 × 75) for each acclimation temperature, and prior to each addition, transitioned to the new acclimation temperature overnight in a separate 300 L tank at a temperature change rate of 0.5 °C per hour. Both species shared one tank but were physically separated by an oyster mesh barrier (Supplementary Fig. S2). Tanks were equipped with two-level oyster mesh dwellings (W × L × H, in cm, 40 × 50 × 50), one for each species, to provide shelter and additional vertical space (Supplementary Fig. S2). Each acclimation tank was filled up to 0.65 m^3^ and supplied with 14.0 °C ([14.0–14.0], *n* = 784,797) cold sea water, temperature controlled via an external heat-chill unit. Additional submersible 2000 W titanium heaters maintained the water temperature in the summer temperature acclimation tanks at 17.5 °C ([17.5–17.5], *n* = 750,404) and 21.4 °C on average ([21.4–21.4], *n* = 775,440) respectively. The temperature for each tank was continuously monitored and logged using open-hardware components^97^, i.e. an open-source microcontroller (Arduino Uno R3, Italy), three waterproof digital temperature probes (DS18B20, China) and a SD card logger module (Adafruit data logger shield, USA), fitted in a custom designed 3D printed plastic enclosure. Two RGB flood lights supplied weak blue light to illuminate acclimation tanks under a 12:12 light:dark photoperiod, controlled remotely via infra-red LEDs and a custom programmed open source microcontroller (Arduino Uno R3, Italy).

Acclimation vessels received filtered and disinfected (sand filtration, foam fractionation, ozonation and activated carbon filtration) flow through sea water at an exchange rate of the tank volume approximately every two hours (flow rate ~ 300 L h^-1^) and regular removal of debris and left-over mussel shells every 2–3 days. Previous testing showed that water quality parameters such as nitrate, ammonia or heavy metals were below critical levels at those water exchange rates.

Animals were fed on Monday, Wednesday, and Friday with live blue mussel (*Mytilus galloprovincial*is) or frozen, chopped sardines (*Sardina pilchardus*). Lobsters that moulted were not used for experiments and allowed to recover for at least 2 weeks. Three southern rock lobsters and two eastern rock lobsters had to be replaced due to excess moulting stress and cannibalism following moulting. At the end of all experiments, we took final measurements of body mass, body volume, carapace length, carapace width, blood pH and Brix index, and returned lobsters to IMAS aquaculture holding tanks.

### Metabolic rate measurements

Once each lobster pair completed its respective acclimation period and was starved for at least 48 h, we performed the following sequence of experiments to measure various aerobic and anaerobic metabolic rate parameters. (1) Exhaustion of lobster in chase tank, (2) measurement of oxygen consumption rate in respirometer, (3) recovery in original acclimation tank, (4) repetition of step 1–3 till each lobster was measured acutely across all three acclimation temperatures (schematic overview in Supplementary Fig. S2). This fully factorial acclimation and acute design, enabled to assess the full range of possible acclimation effects (e.g., partial or complete compensation^98^). Details of each experimental step are outlined below.

Individual lobsters were exhausted to fatigue by manual chasing in a 300 L chase tank (W × L × H, in cm, 120 × 60 × 50) to allow the subsequent measurements of various aerobic- (e.g., maximum metabolic rate) and anaerobic metabolic rate parameters (e.g., excess post-exercise oxygen consumption), in a single respirometry experiment^49,99^. To minimise effects by capture and transfer stress immediately before experiments, each lobster was placed overnight in a covered bucket floating in their original acclimation tank. The next morning, lobsters could be moved directly from the bucket to the chase tank with minimal visual or physical disturbance by the experimenter, to assure lobsters were fully rested before chasing. Due to variable effectiveness of single chasing methods among individuals we applied the following multi-step chase protocol to assure that lobsters were fully exhausted: (1) touching of antennae and (2) gentle pressing of the ventral soft tissue, located between the last pair of pereiopods and the abdomen, to trigger tail flips and (3) lastly turning of the lobster on its back till it failed to turn back within 60 secs three times. Steps (1) and (2) were considered completed till lobsters failed to respond five or more times to the respective procedure. Each chase procedure was video recorded using a camera (GoPro Hero5, GoPro Inc., USA) mounted on top of the chase arena with a flexible gooseneck clamp. A waterproof RGB multi-colour LED strip (5 m 5050 RGB, 60 SMD LEDs/m, Brightness: 900 LM, China) layered 20 cm below the translucent bottom of the chase tank, illuminated the chase arena with yellow light and provided a sharp contrast for the subsequent video analysis. The temperature of the chase tank was set to the experimental temperature using a 2000 W titanium heater modified with a programmable PID (proportional–integral–derivative) controller (SmartPID, Arzaman, Italy). The heating was performed via an external buffer tank, connected to a recirculation pump, to allow an obstruction free chase arena.

Immediately after chasing, we measured the lobster’s oxygen consumption rates (*M*O_2_) using intermittent flow respirometry over a 48–72 h period. For this, we placed lobsters into two cylindrical 10 L custom-made Perspex respirometry chambers (L × D in cm, 66 × 15), tail first to prevent blocking and injury due to the lobster’s guarding posture^100^. Lobsters could gain traction to oyster mesh added to the bottom of the respirometer, held in place with an open cut piece of plastic pipe (see Fig. 7A in Oellermann *et* al. 2020^51^). Chambers were sealed within two minutes after addition of lobsters. Oxygen concentration was measured every 10 s using a fibre optic two-channel oxygen meter (HQ40d, Hach, USA) and oxygen probes positioned into an external recirculation loop. Re-circulation pumps provided continuous mixing of water via Tygon® tubing within respirometers at a flow of 1200 L min^−1^. Following a six-minute respiration cycle, flush pumps re- oxygenated chambers at a flow of ca. 1500 L min^−1^ for eight minutes, using a time controlled digital recycling timer (DRT-1, Sentinel, USA). This assured that oxygen saturation in respiration chambers did not fall below 86.6% on average ([85.8–87.3], *n* = 108), and never below 77%. The two adjacent respirometers were housed in a buffer tank (W × L × H in cm, 102 × 52 × 50), filled with 190 L filtered and filtered flow-through sea water at a flow rate of 130–150 L h^−1^. The water temperature of the buffer tank was maintained at the respective experimental temperature using a 2000 W titanium heater. An air stone ensured homogenous mixing and aeration. Black building foil covering the experimental setup and corrugated plastic sheet between respirometers prevented the lobsters from being visually disturbed during the trials. A yellow LED flood light illuminated the setup permanently to reduce dark-induced activity of the nocturnal lobsters^92^. After completion of respirometry, we returned lobsters to their original acclimation tanks. After each experiment, the chambers and buffer tank were cleaned and flushed with fresh water. Preliminary tests confirmed appropriate mixing of water in the chamber, the lack of leaks (i.e., dye test) and appropriate flush/respiration cycles.

Before the beginning of the next experimental sequence at another experimental temperature, animals were given at least 2 weeks’ time between experiments to recover in their original acclimation tanks. In addition, experiments with a large difference between acclimation- and experimental temperature (e.g., 14.0 °C 21.5 °C) were performed last and included a short, stepwise acclimatization period to the final experimental temperature for at least three hours prior to measurements to reduce acute temperature stress.

### Calculations and analysis

Oxygen consumptions rates were calculated as in Svendsen et al.^101^. Individual body volume of lobsters was accounted for in *M*O_2_ calculations and measured as the volume overflow in ml after adding lobsters to a water levelled container. Body density did not differ significantly between species (ANOVA, *F*_(1,45)_ = 2.31, *p* = 0.136) and averaged 1.12 g ml^−1^ ([1.10–1.14], *n* = 46). Background respiration was recorded before and after each experiment and accounted on average for 4.5% ([3.9–5.1], *n* = 105) or 5.0% ([4.3–5.6], *n* = 105) respectively of standard metabolic rate. Maximum metabolic rate (MMR) was the single highest *M*O_2_ measured over the entire experiment, and routine metabolic rate the mean *M*O_2_ following a 16 h recovery period till the end of the trial (16 h was observed to be the maximum time all lobsters required to recover). Standard metabolic rate was calculated as the mean of the 10% lowest *M*O_2_ values^34,99^. The time at which *M*O_2_ fell three times below routine metabolic rate + one standard deviation after exercise, was marked as recovery time. Excess post-exercise oxygen consumption (EPOC) was calculated as the area under the *M*O_2_ curve from the start of *M*O_2_ measurements till recovery time. Aerobic scope was calculated as maximum metabolic rate—standard metabolic rate^34,99^. Recovery rate was calculated as EPOC divided by recovery time and expressed as mg O_2_ per kg body mass and hour.

We analysed the chase videos using a customised Python script (Python 3.8, Supplementary File S1) to record total number of escapes and escape speed. Here we marked individual lobster positions (by mouse clicks) before and after each tail flip and saved the x and y pixel positions and the respective video frame number in a csv file. All further data processing and statistical data analysis was performed using R statistical software^102^ and RStudio^103^. In R we calculated the Euclidian distance Eq. (1) and divided this by the number of video frames for each corresponding escape event, times the recording frame rate (24 frames sec^−1^) and a conversion factor to scale pixels to cm to obtain the escape speed (cm sec^−1^, R scripts in Supplementary Files S2).

1 $${\text{Euclidian distance}}:d = \sqrt {\mathop \sum \limits_{i = 1}^{v} (p_{1i} - } p_{2i} )^{2}$$

For the statistical analysis we employed linear mixed effect models (LME) to test the effects of acclimation- and experimental temperatures on each of the measured variables (maximum metabolic rate, aerobic scope, fractional aerobic scope, EPOC, recovery time, escape speed and total escapes) using the *lme4* package^104^. Detailed model outputs and effect sizes for main effects were summarised in Supplementary Table S2. For all models, we initially included acclimation temperature, experimental temperatures and species and their three-way interaction as well as sex and body mass as fixed factors. Lobster ID was included as random factor to account for the repeated measurement of individuals at different experimental temperatures and inter-individual variation. We first computed this complex model for each response variable using maximum likelihood estimation (ML), and then identified the simplest model using a stepwise backward elimination process of fixed factors, via Satterthwaite's approximation of *p*-values, using the *lmerTest* package^105^. We then re-fitted the final model using restricted maximum likelihood (REML) estimation, tested the effects of the main factors of the final model using ANOVA, and performed post hoc pairwise comparisons with Bonferroni correction between levels of acclimation temperature and experimental temperature using the *emmeans* package^106^. We assessed the linearity, homoskedasticity and normality of residuals using residual plots and the Shapiro–Wilk normality test respectively, to test if the data meet the linear mixed effect model assumptions. Because standard metabolic rate followed a non-normal distribution, we fitted a generalised linear mixed effect model (GLMM) with a gamma distribution and an identity link function using the glmmTMB R package^107^. Fixed factors that did not improve the GLMM model further were dropped if the difference between the model’s Akaike Information Criteria (ΔAIC) was > 2 (i.e., species and body mass), while retaining experimental- and acclimation temperature and sex as fixed factors and animal ID as random factor in the final GLMM model (Table 2). Detailed GLMM model results were summarised in Supplementary Table S3. Relations between escape metrics and EPOC or maximum metabolic rate were tested separately for each species using Pearson correlation, if data passed the Shapiro–Wilk normality test. All values in the manuscript are expressed as mean and the 95% confidence interval in squared brackets. In-text results of the main factor effects of the final linear mixed effect models using ANOVA were reported as: *F*_(df)_ = *F* value, *p* = Pr(> *F*). Final mixed effect models for all response variables are listed in Table 2 and Supplementary Table S2. Complete analysis and model calculations are available as R Markdown files in the Supplementary Files S2. The complete data set can be found in Supplementary Table S1.

## Supplementary Information

Supplementary Information 1.

Supplementary Information 2.

## Data Availability

The dataset generated and analysed during the current study and all supplementary information are available for download in the figshare repository: 10.6084/m9.figshare.15134241.
