# Supplementary material for: Metabolic plasticity improves lobster’s resilience to ocean warming but not to climate-driven novel species interactions
Source: Sci Rep. 2022 Mar 15;12:4412. doi: 10.1038/s41598-022-08208-x (PMC8924167; doi:10.1038/s41598-022-08208-x)
Supplement: Supplementary file 1 — Supplementary Information 1. [file 41598_2022_8208_MOESM1_ESM.docx]

**Supplementary information**

**Research Article:**

**Metabolic plasticity improves lobster´s resilience to ocean warming but not to climate-driven novel species interactions**

Michael Oellermann, Quinn P. Fitzgibbon, Samantha Twiname, Gretta T. Pecl

**Supplementary Figure S1:** Oxygen consumption rate of moulting eastern rock lobster *S. verreauxi*, following exhaustive exercise and a recovery period. Moulting caused the exhaustion of the full aerobic scope and an elevation of post-moulting oxygen consumption above standard metabolic rate.


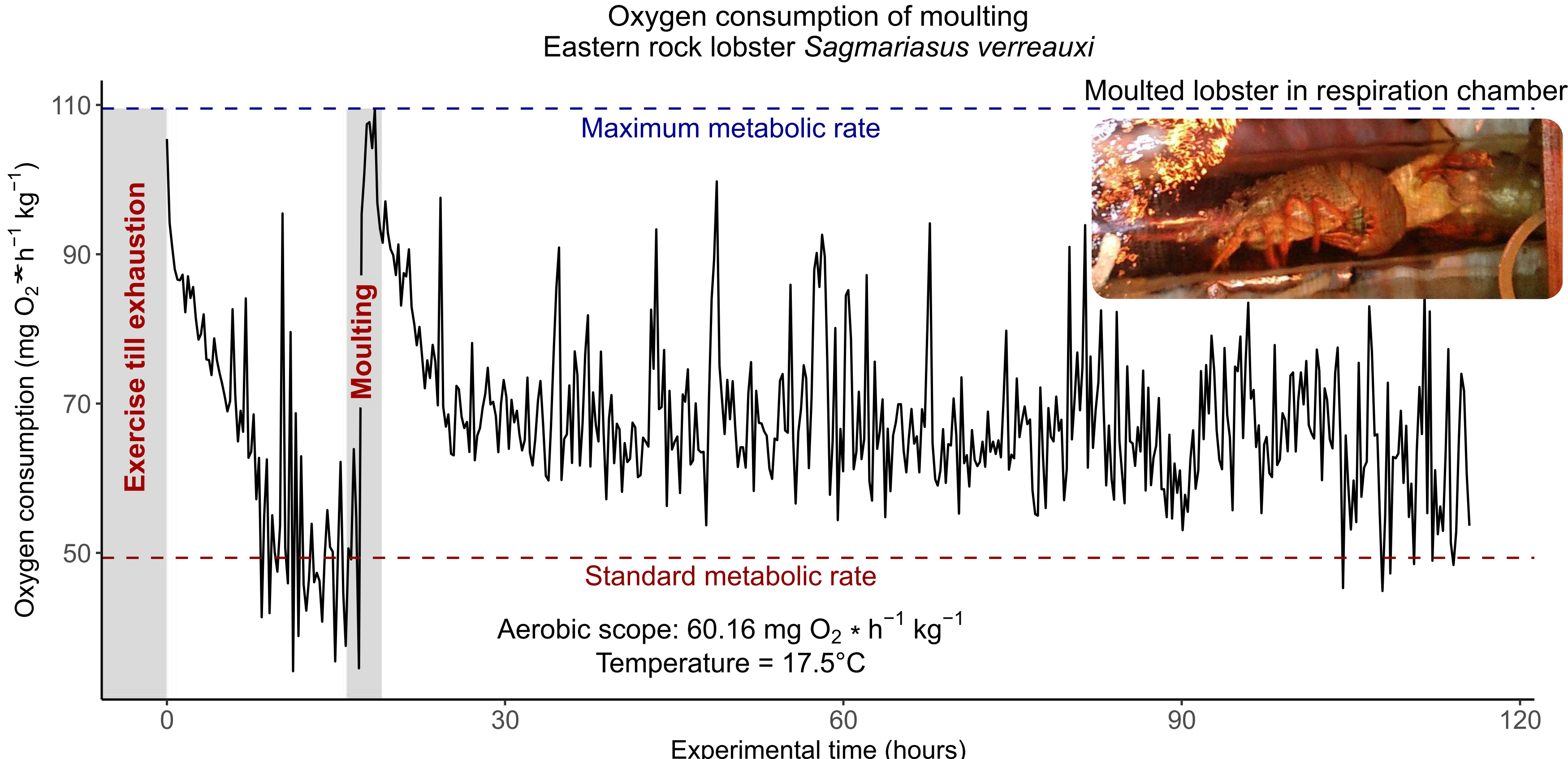


**Supplementary Figure S2:** Schematic diagram of the fully factorial experimental design. Eastern and southern rock lobsters were acclimated in pairs to current average (14°C), current summer (17.5°C) and future summer temperatures (21.5°C) for at least eight weeks. To detect differences between acclimation treatments, we measured oxygen consumption rates of each lobster, across all three acclimation temperatures, using intermittent respirometry.
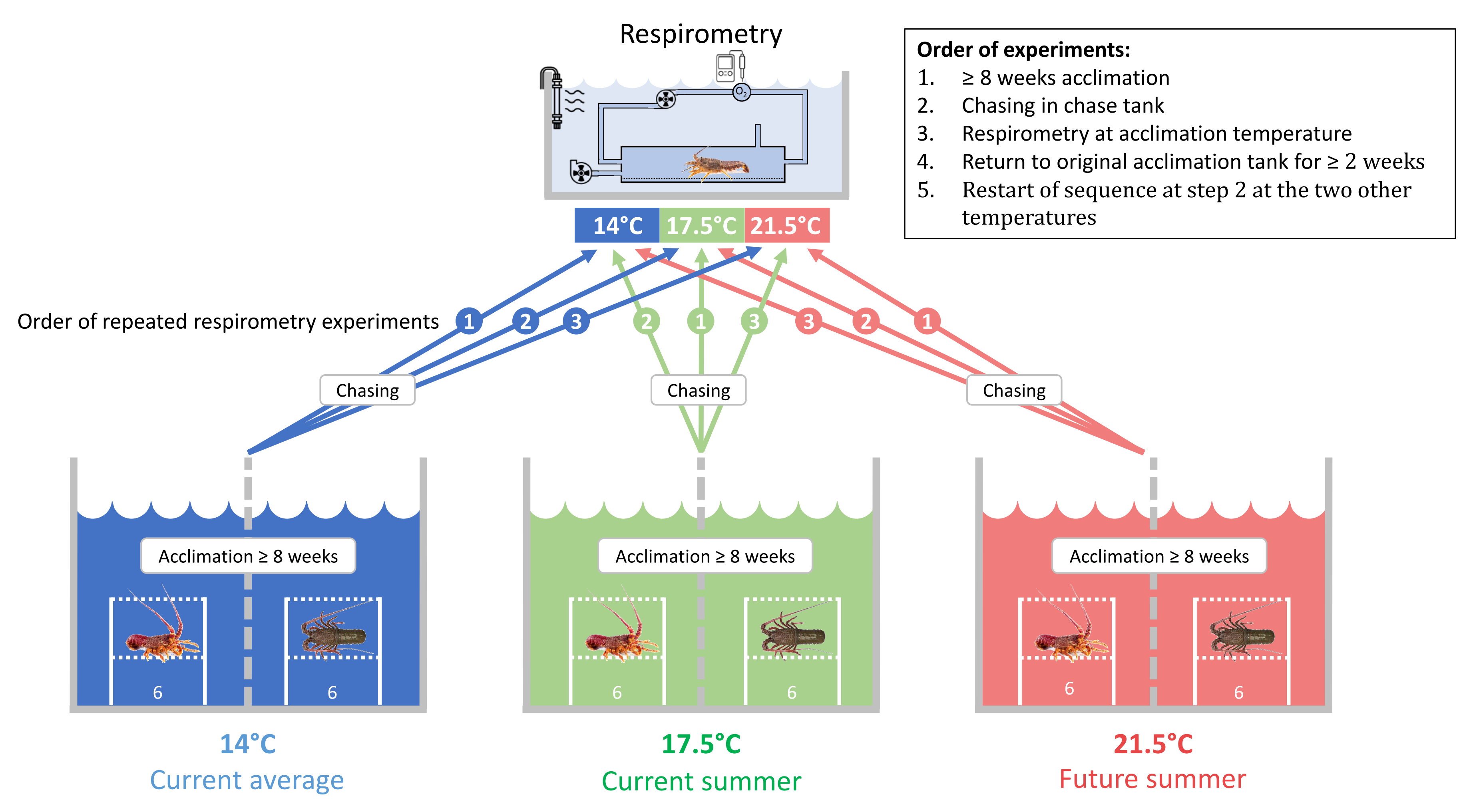


**Supplementary Table S1:** Raw data table for all individuals used in this study available in the figshare repository ([LINK](https://figshare.com/s/cbed7c33a3c57c3950b3)).

**Supplementary File S1:** Python script to record total escapes and escape speed available in the figshare repository ([LINK](https://figshare.com/s/cbed7c33a3c57c3950b3)).

**Supplementary Files S2:** R Markdown files for data processing and statistical analysis available in the figshare repository ([LINK](https://figshare.com/s/cbed7c33a3c57c3950b3)).

**Supplementary Files S3:** R Markdown file for weight and sex distribution of animals available in the figshare repository ([LINK](https://figshare.com/s/cbed7c33a3c57c3950b3)).

**Supplementary Table S2:** Summary of the impact of main effects on various metabolic performance and escape parameters of Southern- and Eastern rock lobster using linear mixed effect models. Effect sizes of *η*^2^ ≤ 0.06 indicate a small effect; *η*^2^ > 0.06 < 0.14 a medium effect; and *η*^2^ ≥ 0.14 a large effect. Main effects with *p* ≤ 0.05 were shaded grey.

| **Factor** | **Main Effect** | ***F* (df)** |  | **Significance (*p*-value)** | **Partial Effect Size (η^2^_p_ [C.I. 90%])** | **Effect Size Category** |
| --- | --- | --- | --- | --- | --- | --- |
| **Maximum metabolic rate** | Experimental Temp. | 59.71 (2) |  | 7.446 x 10^-15^ | 0.67 [0.55-0.75] | large |
|  | Acclimation Temp. | 3.1 (2) |  | 5.913 x 10^-15^ | 0.17 [0.00-0.34] | large |
|  | Species | 9.54 (1) |  | 0.004 | 0.23 [0.05-0.43] | large |
|  | Body mass | 4.13 (1) |  | 0.050 | 0.10 [0.00-0.28] | medium |
|  | Experimental x Acclimation Temp. | 2.37 (4) |  | 0.062 | 0.14 [0.00-0.27] | medium |
|  | Experimental Temp. x Species | 2.49 (2) |  | 0.091 | 0.08 [0.00-0.19] | medium |
|  | Acclimation Temp. x Species | 1.05 (2) |  | 0.361 | 0.06 [0.00-0.21] | medium |
|  | Experimental x Acclimation Temp. x Species | 0.45 (4) |  | 0.775 | 0.03 [0.00-0.07] | small |
| **Aerobic scope** | Experimental Temp. | 1.88 (2) |  | 0.161 | 0.05 [0.00-0.15] | small |
|  | Acclimation Temp. | 0.59 (2) |  | 0.562 | 0.03 [0.00-0.14] | small |
|  | Species | 5.85 (1) |  | 0.021 | 0.15 [0.01-0.33] | large |
|  | Experimental x Acclimation Temp. | 3.62 (4) |  | 0.010 | 0.18 [0.03-0.29] | large |
| **Factorial aerobic scope** | Experimental Temp. | 44.51 (2) |  | 2.929 x 10^-13^ | 0.56 [0.43, 0.65] | large |
|  | Acclimation Temp. | 3.86 (2) |  | 0.03 | 0.17 [0.01, 0.34] | large |
| **EPOC** | Experimental Temp. | 18.07 (2) |  | 7.102 x 10^-7^ | 0.37 [0.21-0.50] | large |
|  | Acclimation Temp. | 10.93 (2) |  | 2.298 x 10^-4^ | 0.40 [0.17-0.56] | large |
|  | Species | 2.02 (1) |  | 0.165 | 0.06 [0.00-0.22] | small |
|  | Experimental x Acclimation Temp. | 2.77 (4) |  | 0.035 | 0.16 [0.01-0.26] | large |
|  | Experimental Temp. x Species | 0.15 (2) |  | 0.859 | 0.01 [0.00-0.04] | small |
|  | Acclimation Temp. x Species | 0.27 (2) |  | 0.763 | 0.02 [0.00-0.10] | small |
|  | Experimental x Acclimation Temp. x Species | 2.47 (4) |  | 0.054 | 0.14 [0.00-0.25] | large |
| **Recovery time** | Experimental Temp. | 6.64 (2) |  | 0.002 | 0.16 [0.04-0.28] | large |
|  | Acclimation Temp. | 10.11 (2) |  | 3.369 x 10^-4^ | 0.36 [0.14-0.52] | large |
|  |  |  |  |  |  |  |
| **Supplementary Table S2 continued** | |  |  |  |  |  |
|  |  |  |  |  |  |  |
| **Recovery rate** | Experimental Temp. | 16.95 (2) |  | 1.167 x 10^-6^ | 0.34 [0.18-0.47] | large |
|  | Acclimation Temp. | 2.61 (2) |  | 0.088 | 0.13 [0.00-0.29] | large |
|  | Experimental x Acclimation Temp. | 4.84 (4) |  | 0.002 | 0.23 [0.06-0.34] | large |
| **Escape speed (cm s^-1^)** | Experimental Temp. | 5.23 (2) |  | 0.008 | 0.14 [0.02-0.27] | large |
|  | Species | 11.85 (1) |  | 0.002 | 0.26 [0.07-0.45] | large |
| **Total escapes** | Species | 8.24 (1) |  | 0.007 | 0.20 [0.04-0.38] | large |
|  | Body mass | 3.72 (1) |  | 0.061 | 0.09 [0.00-0.26] | medium |

**Supplementary Table S3:** Summary of the impact of main effects on standard metabolic rate of Southern- and Eastern rock lobster using general linear mixed effect models.

| **Factor** | **Main effect** | **Beta [C.I. 95%]** | **Significance (p-value)** |
| --- | --- | --- | --- |
| SMR | Experimental Temp. (21.5°C) | 26.97 [21.26, 32.68] | 2.0 x 10^-16^ |
|  | Acclimation Temp. (21.5°C) | -5.01 [-8.33, -1.70] | 0.003 |
|  | Sex (male) | -2.62 [-5.22, -0.02] | 0.048 |
|  | Experimental (21.5°C) x Acclimation Temp. (21.5°C) | -10.48 [-17.28, -3.68] | 0.003 |
